# Molecular screening of cancer-derived exosomes by surface plasmon resonance spectroscopy

**DOI:** 10.1007/s00216-015-8711-5

**Published:** 2015-04-30

**Authors:** Luigino Grasso, Romain Wyss, Lorenz Weidenauer, Ashwin Thampi, Davide Demurtas, Michel Prudent, Niels Lion, Horst Vogel

**Affiliations:** Laboratory of Physical Chemistry of Polymers and Membranes, Ecole Polytechnique Fédérale de Lausanne, Station 6, 1015 Lausanne, Switzerland; Interdisciplinary Center for Electron Microscopy, Ecole Polytechnique Fédérale de Lausanne, Station 12, 1015 Lausanne, Switzerland; Transfusion Interrégionale CRS, Laboratoire de Recherche sur les Produits Sanguins, Rte de la Corniche 2, 1066 Epalinges, Switzerland

**Keywords:** Exosome, Surface plasmon resonance, Cancer, Blood, Biomarker, Screening

## Abstract

We report on a generic method to detect and identify the molecular profile of exosomes either derived from cultured cell lines or isolated from biofluids. Exosomes are nanovesicles shed by cells into their microenvironment and carry the molecular identity of their mother cells. These vesicles are actively involved in intercellular communication under physiological conditions and ultimately in the spread of various diseases such as cancer. As they are accessible in most biofluids (e.g., blood, urine, or saliva), these biological entities are promising tools for cancer diagnostics, offering a non-invasive and remote access to the molecular state of the disease. The composition of exosomes derived from cancer cells depends on the sort and state of the tumor, requiring a screening of multiple antigens to fully characterize the disease. Here, we exploited the capacity of surface plasmon resonance biosensing to detect simultaneously multiple exosomal and cancer biomarkers on exosomes derived from breast cancer cells. We developed an immunosensor surface which provides efficient and specific capture of exosomes, together with their identification through their distinct molecular profiles. The successful analysis of blood samples demonstrated the suitability of our bioanalytical procedure for clinical use.

**Electronic supplementary material:**

The online version of this article (doi:10.1007/s00216-015-8711-5) contains supplementary material, which is available to authorized users.

## Introduction

Molecular diagnostics aims to assess the pathophysiological conditions of the patient by primarily targeting both nucleic acids and proteins. Alterations in protein expression or function together with nucleic acid mutations or copy number changes are the principal molecular indicators of a disease. Identifying these biomarkers in cancer patients can provide precious information on both the prognosis of the disease and the predictive outcome of a given therapy [1]. Generally, a single biomarker will not be sufficient for a reliable diagnosis as cancer is a highly heterogeneous disease with multiple stages and subtypes reflected by multiple molecular abnormalities [2, 3]. Hence, a set of biomarkers describing both the genome and proteome is fundamental to fully characterize the tumor profile and pave the way for the development of a highly specific and reproducible diagnostic test [4]. In that context, single exosomes are promising candidates as they transport the molecular identity of their mother cells. These nanometer-sized containers of endosomal origin are released by cells through the fusion of multivesicular bodies with the plasma membrane. This secretion is modulated by environmental stress leading to concentration variation in body fluids such as blood, urine, or breast milk [5]. Exosomes play a central role in the intercellular communication and, thereby, also in the propagation of diverse pathologies, *in primis* cancer [6] and inflammatory [7] and neurodegenerative [8] diseases, by activating signaling cascades in/or delivering bioactive molecules such as lipids, proteins, or RNAs to the recipient cells. Exosome isolation followed by bioanalysis would enable a non-invasive and remote biopsy of the tumor mass. Considerable efforts are presently undertaken to identify and screen exosomal cell surface receptors with the help of diverse analytical tools. For example, proteomic analysis is mainly performed by immunoassays [9] and mass spectrometry [10], which are time-consuming techniques requiring large amount of exosomes and are thus not suitable for clinical applications. On the other hand, surface-sensitive techniques have emerged as powerful and robust tools for characterizing biomolecular interactions [11–13]. A striking example is the surface plasmon resonance (SPR) biosensor which has become a gold standard for the real-time and label-free monitoring of biomolecular interactions [14, 15]. A SPR biosensor exploits the evanescent surface plasmon wave at the surface of a gold layer to probe the optical properties of the contacting dielectric region and thereby quantifies changes in the number of biomolecules at the sensor surface induced by molecular interactions. Selective functionalization of the sensor gold surface with capturing agents (e.g., antibodies, ligands, and nucleic acids) enables the determination of the thermodynamics (binding constants) and kinetics (rate constants) of specific molecular interaction reactions. The versatility of SPR allows the measurement of a broad range of biological targets for various applications. For instance, SPR has been extensively used for high-throughput screening of biological active compounds [16], discovering novel biomolecular interactions [17] and investigating dynamic processes involved in signaling pathways [18, 19]. Moreover, complex biological fluids (e.g., blood and urine) are compatible with SPR which renders this technique attractive for quantitative analyses in clinical laboratories [20], such as for Alzheimer’s disease [21], viruses [22], infection-related antibodies [23], and cancer [24–26]. Only recently SPR has shown its potential in the field of exosome research. Exosome concentration and screening of membrane proteins were determined with commercial SPR instruments [27, 28], while a larger molecular profiling of exosomes derived from both culture cell lines and cancer patients has been performed with a miniaturized SPR device based on nano-plasmonic holes [29]. In a continuation of these studies, we have implemented this technique in the particular clinical case of breast cancer. We have analyzed by SPR exosomes isolated from three cultured cell lines of human breast cancer, namely MCF-7, BT-474, and MDA-MB-231, each of these cell lines representing a different class of breast carcinoma, luminal A, luminal B, and claudin-low, respectively [30]. The detection of the relative low concentration of the biomarkers was only possible after we developed a suitable sensor surface, drastically reducing nonspecific binding while optimizing the sensitivity and stability for the specific signal. Various cancer and exosomal biomarkers were targeted, leading to a characteristic molecular signature for each cell line. For assessing the clinical applicability and usability of our biosensor, we analyzed exosomes isolated from whole blood. The methodology is readily implementable in any academic or clinical laboratory and would provide a unique fingerprint of the status of a particular disease based on the exosomal protein expression pattern, paving the way for a novel breast cancer diagnostic tool.

## Materials and methods

### Materials

Dulbecco’s phosphate-buffered saline (D-PBS, Sigma), DMEM/F-12+ GlutaMAX (Life Technologies), Newborn Calf Serum (NBCS, Life Technologies), α-mercapto-ω-carboxy-PEG3000 (long PEG, *n* = 68, Sigma), PEG acid disulfide (short PEG, *n* = 7, Sigma), neutravidin (Thermo Scientific), *N*-ethyl-*N*′-(3-dimethylaminopropyl)carbodiimide hydrochloride (EDC, Sigma), *N*-hydroxysuccinimide (NHS, Sigma), ethanolamine (Sigma), and sodium dodecyl sulfate (SDS, Sigma).

### Cell culture

MDA-MB-231, MCF-7, and BT-474 human breast cancer cells (kind gift from Prof. C. Brisken, EPFL, Switzerland) were grown in medium supplemented with 10 % NBCS (unless otherwise stated) in a humidified 5 % CO_2_ atmosphere at 37 °C.

### Isolation of exosomes

Exosomes from cultured breast cancer cell lines were isolated using a previously published protocol [31] with slight modifications. Briefly, the conditioned medium (CM) of ∼40 × 10^6^ cultured cells, maintained in serum-free medium during the last 48 h, was first centrifuged at 300*g* for 4 min and then filtered through a 0.22-μm pore-sized filter. Exosomes were concentrated by ultrafiltration (UF) using a 100-kDa molecular weight cutoff (MWCO) Amicon Ultra-15 centrifugal filter unit (Millipore) resuspended in D-PBS and concentrated again by UF to a volume of approximately 250 μl. Exosomes were further purified by size exclusion chromatography (SEC) using a Sephacryl 500 10/40 GL column (GE Healthcare) equilibrated in D-PBS with an ÄKTA-Purifier system (GE Healthcare). Exosome-containing fractions were identified by absorption at *λ* = 280 nm and concentrated to the desired volume with a 100-kDa MWCO Amicon Ultra-4 centrifugal filter unit (Millipore). For exosome isolation from clinical samples, plasma was obtained from healthy volunteers from the Laboratoire de Recherche sur les Produits Sanguins, Lausanne, Switzerland. Five hundred microliters of plasma was centrifuged at 300*g* for 4 min, filtered through a 0.22-μm pore-sized filter, and then purified by SEC using the aforementioned procedure.

### Size distribution of exosomes by DLS

Experiments were carried out on a Zetasizer Nano ZS (Malvern) equipped with a He–Ne laser of 633 nm (4 mW), and the light scattering from the samples was measured at an angle of 175° to the incident laser beam. A typical experiment comprised a sequence of 12 × 10 s recordings which were repeated three times.

### Cryo-EM

Exosomes were processed for visualization by cryo-EM as previously described [31].

### Molecular screening by SPR

Experiments were carried out on a Biacore 3000 instrument (GE Healthcare) with D-PBS as running buffer at a flow rate of 5 μl/min, unless otherwise stated. The gold-coated sensor surfaces (SIA Kit AU, GE Healthcare) were first cleaned in piranha solution (H_2_SO_4_/H_2_O_2_, 3:1) for 30 min at 50 °C, rinsed extensively with distilled water and ethanol, and finally dried under a stream of nitrogen. A self-assembled monolayer of carboxylated polyethylene glycol (PEG) polymers was then formed on the gold surfaces by overnight immersion in an ethanolic solution containing 2 mM of 99 mol % short PEG and 1 mol % long PEG. Unbound PEGs were removed by 1 min sonication in fresh ethanol. The gold surface was then dried under a stream of nitrogen, mounted on the sensor chip support, and docked into the Biacore 3000. Neutravidin was covalently coupled to carboxylated PEGs using standard amine coupling procedure by injecting the reagents in the following order: 35 μl 0.05 M NHS/0.2 M EDC mixture, 60 μl neutravidin at 100 μg/ml in 10 mM acetic acid (pH 4.5), and 35 μl 0.1 M ethanolamine. Unbound proteins were removed upon injection of 4× 100 μl SDS 0.05 % at 100 μl/min. The amount of immobilized neutravidin was approximately 2500 RU. Biotinylated antibodies (Electronic Supplementary Material (ESM) Table S1) were diluted in D-PBS at a concentration of 0.05 mg/ml, and 15 μl of these solutions was injected over the neutravidin-coated surface, reaching typical immobilization levels of 2000 RU. Exosome solutions were finally injected sequentially for 4 min over the antibody-functionalized surface. BiaEvaluation Software Version 4.1 (Biacore) and IGOR Pro Version 6.34A (Wavemetrics) were used for data processing. Sensorgrams were corrected for bulk refractive index changes and nonspecific binding by subtracting the negative control signal. The molecular fingerprints were obtained by weighting maximal responses with the respective antibody surface coverage and normalized to anti-CD63 responses.

### Molecular screening by ELISA

Exosomes were directly coated on the surface of a 96-well plate (Greiner). Fifty microliters/well of purified exosome solution was added and incubated overnight at 4 °C. The plate was washed with D-PBS and blocked with 100 μl/well of blocking solution (D-PBS containing 5 % BSA) at room temperature for 1.5 h. Following three washes of D-PBS, 50 μl/well of primary antibodies at 2 μg/ml in blocking solution were added for 1 h at room temperature (RT). After three washes of D-PBS, the plate was incubated with 50 μl/well of HRP-conjugated anti-mouse IgG antibody (Sigma, 1:10,000 dilution) in blocking solution for 1 h at RT. The wells were finally washed three times. HRP-bound antibodies were detected by adding 100 μl of 3,3′,5,5′-tetramethylbenzidine (TMB, Sigma) solution, and the reaction was stopped with 100 μl 2 M H_2_SO_4_ after 30 min. The optical densities were recorded at 450 nm using the multiwell plate reader SpectraMax 360 (Molecular Devices).

## Results and discussion

### Purification and characterization of exosomes

**Fig. 1:**
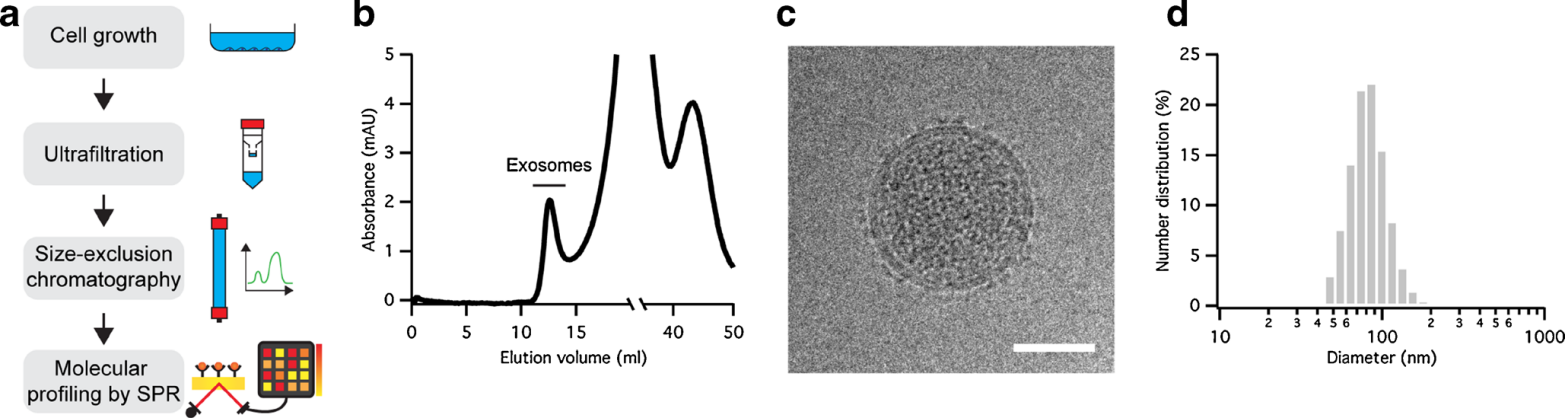
Purification of exosomes derived from breast cancer cell lines. **A** Typical workflow for the isolation, enrichment, purification, and analysis of exosomes from conditioned medium. The combination of UF and SEC produces ultrapure upconcentrated samples of exosomes. Screening of exosomes across a panel of antibodies is performed by SPR. **B** SEC elution profile of an ultrafiltrated CM of MCF-7 cells. The absorbance was measured at 280 nm, reflecting the amount of protein content. The exosomal fraction appeared between 11 and 14 ml elution volume. **C** Cryo-EM micrograph of an exosome secreted by MCF-7 cells. Scale bar 50 nm. **D** Size distribution of MCF-7 exosomes obtained by DLS

Exosomes shed by the three breast cancer cell lines, MCF-7, BT-474, and MDA-MB-231, were isolated from cell debris and large particles by filtration using a 0.22-μm pore-sized filter and subsequently concentrated by ultrafiltration (UF) over a 100-kDa molecular weight cutoff filter (Fig. 1a). To ensure ultrapure samples free of any residual soluble proteins, the exosomes were further purified by size exclusion chromatography (SEC). A typical elution profile is depicted in Fig. 1b, showing a small but well-resolved peak around 12.5 ml elution volume originating from the optical absorption at 280 nm of exosomal proteins. Soluble, non-exosomal proteins are eluted at larger elution volumes as broader peaks. Cryo-electron microscopy (cryo-EM) micrographs revealed well-preserved purified exosomal vesicles with closed circular lipid bilayers comprising densely packed membrane proteins and clean of any large contaminants (Fig. 1c). The size distribution of the purified vesicles, determined by dynamic light scattering (DLS), ranged from 30 to 200 nm (Fig. 1d), in good agreement with data published elsewhere [31].

### Functionalization of the immunosensor surface

**Fig. 2:**
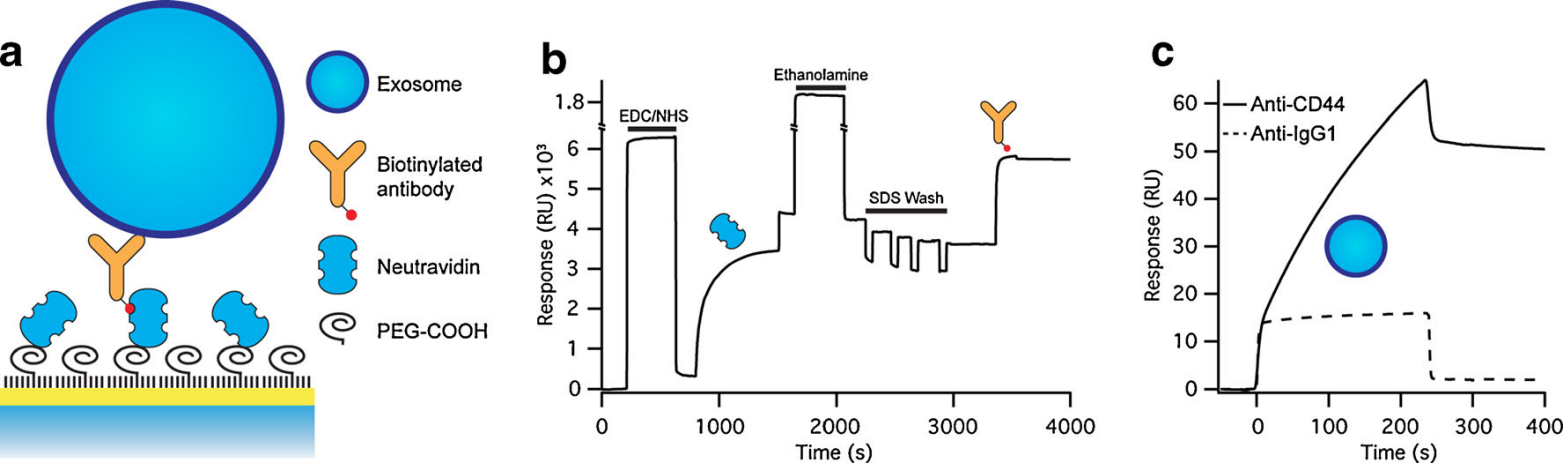
SPR analysis of exosomes. **A** Scheme of the functionalization of the SPR sensor surface. The sensor surface was first passivated with a mixture of short and long carboxylated PEGs to which neutravidin was covalently bound, followed by functionalization with biotinylated antibodies. **B** Typical SPR sensorgram of the sensor chip functionalization. Neutravidin is attached to the surface using standard amine coupling chemistry to subsequently capture the biotinylated antibodies. **C** Specific detection of MCF-7 exosomes with biotinylated anti-CD44 antibodies (*solid line*). Anti-rat IgG1 was used as a negative control (*dashed line*)

To characterize the molecular identity of the biomarkers on the exosomal membrane, the SPR sensor surface must specifically bind the target analyte and sustain a stable signal. Our surface modification strategy is based on: (i) passivation with polyethylene glycol (PEG) polymers, which efficiently suppress nonspecific binding [32], and (ii) functionalization with neutravidin, which forms on the sensor surface a strong and long-lasting complex with biotinylated antibodies [33] (Fig. 2a). The gold surface was first modified with a self-assembled monolayer composed of a 99:1 M ratio of short and long carboxylated PEGs which drastically reduced the nonspecific binding (Fig. 2c). After activation of the surface-accessible carboxyl groups and the subsequent covalent attachment of the amino groups of neutravidin, the sensor surface was functionalized with biotinylated antibodies (Fig. 2b and ESM Table S1). This functionalization procedure is ideally suited to produce a stable and robust baseline signal with no loss of capturing agents. The thus prepared sensor surface efficiently captures exosomes with high specificity as demonstrated by the negligible response (<3 %) for a control antibody (Fig. 2c). This surface modification combined with the microfluidic system of the SPR instrument made it possible to test a panel of capturing agents in parallel with minimum sample volumes (20 μl). Thereafter, we screened the exosomes for the presence of two exosomal markers, CD63 and CD9, which are transmembrane proteins of the tetraspanin family [34] and four cancer markers CD24, CD44, epithelial cell adhesion molecule (EpCAM), and human epidermal growth factor receptor 2 (HER2). The progression of many tumors are reflected in a modification of the expression of CD44 versus CD24 [35, 36], while EpCAM and HER2 are often expressed preferentially in breast cancer cells and are therefore used to subdivide breast cancer into subtypes to fine-tune therapies [37].

### Molecular screening of exosomes

**Fig. 3:**
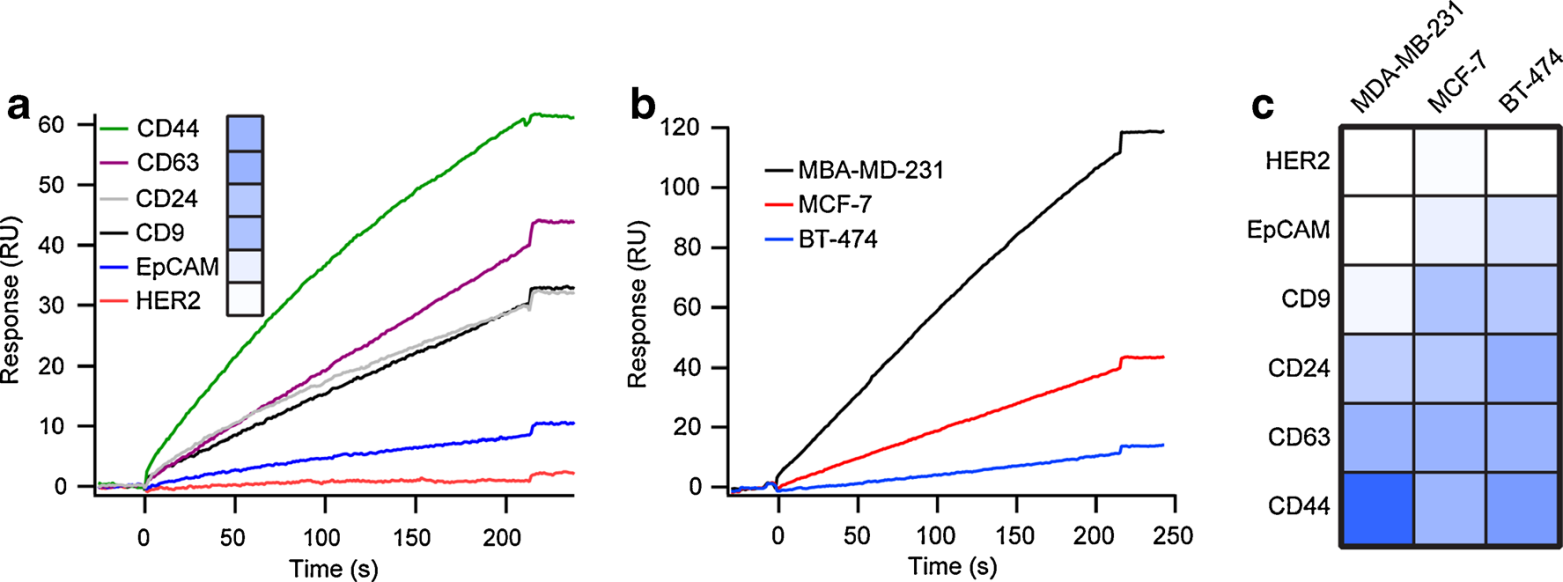
Molecular screening of exosomes with immunosensor chips. **A** Multiplexed analysis of MCF-7 exosome membrane proteins by SPR. The sensorgrams display the capture of exosomes with a panel of antibody: anti-CD63, anti-CD9, anti-CD44, anti-CD24, anti-EpCAM, and anti-HER2. The *inset* shows the relative expression profile of the antigens. **B** Specific binding of MDA-MB-231, MCF-7, and BT-474 exosomes to anti-CD63 antibody. **C** Molecular profiling of breast cancer and exosomal biomarkers in exosomes derived from the three breast cancer cell lines, MDA-MB-231, MCF-7 and BT-474. Sensorgrams in **A** and **B** were corrected for bulk refractive index changes and nonspecific binding

We first performed our molecular screening on exosomes shed by MCF-7 cells, knowing that this particular cell line is representative of the breast cancer subtype luminal A [30]. Typical MCF-7 exosomes binding on the immunosensor chip are presented in Fig. 3a. The observed variation in responses across the panel of antibodies provides evidence of the capacity of our sensor to detect and quantify proteins on the surface of exosomes. The expression profile of the corresponding antigens was obtained by taking into consideration the respective antibody surface coverage and assuming similar binding affinities of the different antibodies (inset of Fig. 3a). Interestingly, exosomal markers, CD63 and CD9, and cancerous markers, CD44 and CD24, exhibited a similar expression level while EpCAM was significantly less abundant on the surface of exosomes. On the opposite, antibodies anti-HER2 were totally inefficient to capture exosomes as expected by the molecular pattern of this breast cancer cell line [30]. These observations were confronted with those obtained with standard enzyme-linked immunosorbent assay (ELISA, ESM Figure S1A), which provided a comparable molecular profile (ESM Figure S2A). However, we note a discrepancy for CD24 which might be explained by the lack of surface coverage control inherent to ELISA. Moreover, ELISA required numerous handling steps and incubation times which delay the analysis and might increase the variability among and within samples. As exosomes of different origins presumably vary in their membrane protein expression profile, we tested two other breast cancer cell lines as a source of exosomes, MDA-MB-231 and BT-474. SPR can provide precise information on the exosome production and concentration through the responses obtained with ubiquitous exosome markers [27, 29]. Hence, the signal arising from exosome binding to anti-CD63 antibodies was used here as a real-time standard calibration reflecting the quantity of injected exosomes, circumventing any errors originating from batch-to-batch variation (Fig. 3b). The various responses between the different exosomes and the immunosensor surfaces formed specific molecular signatures which are graphically represented in the color-coded table of Fig. 3c; CD63 response was used as a normalization factor between exosomes preparations. Exosomes derived from MDA-MB-231 cells were observed to be enriched in CD44 molecules which reflect their cellular origin. Surprisingly, antibodies against HER2 failed to capture exosomes shed by BT-474 cells, a member of luminal B cancer subtype and known to express HER2. This demonstrates either a poor affinity of this antibody against this receptor or ultimately the exclusion of this antigen from the surface of exosomes. Importantly, surfaces functionalized with antibodies against EpCAM successfully discriminate exosomes derived from MDA-MB-231 from those isolated from MCF-7 and BT-474. This result corroborates previous observations where MCF-7 and BT-474 cells were shown to express more of this antigen [38] and demonstrates the effectiveness of our molecular profiling.

### Analysis of blood samples

**Fig. 4:**
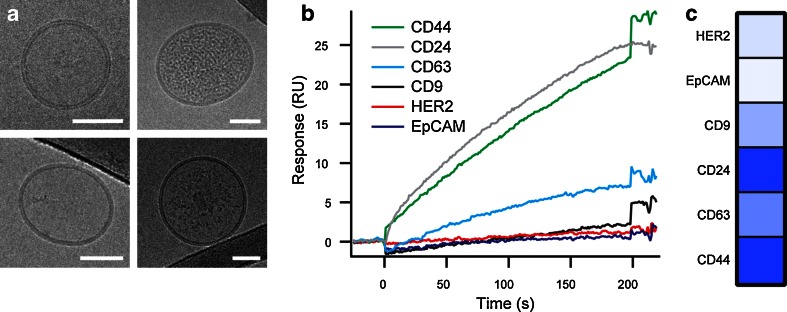
Characterization and molecular analysis of exosomes isolated from human blood plasma. **A** Four cryo-EM micrographs of purified exosomes. Scale bar, 50 nm. **B** Multiplexed SPR analysis of membrane proteins in exosomes. The sensorgrams display the capture of exosomes with a panel of antibody: anti-CD63, anti-CD9, anti-CD44, anti-CD24, anti-EpCAM, and anti-HER2. Sensorgrams were corrected for bulk refractive index changes and nonspecific binding. **C** Relative expression profile of the antigens on the surface of plasma exosomes

The clinical applicability and usability of our procedure, which consists of exosome purification and antibody screening, were assessed by analyzing plasma samples obtained from healthy donors. Since exosomes are naturally found in blood in very high numbers (10^8^–10^11^ per mL) [9, 39, 40], 0.5 mL of blood plasma contains sufficient exosomes to perform a complete screening of antibodies by SPR. We used the aforementioned isolation protocol, except that the plasma was not concentrated by UF. Briefly, the plasma was first centrifuged and filtered to remove cells, cell debris, and large particles and then injected into the SEC column. Compared to classical procedures using differential ultracentrifugation [41], this methodology offers several advantages for the isolation of exosomes from biofluids, especially from blood derived samples: (i) the purified exosomes retain their morphological integrity without aggregating as demonstrated by cryo-EM (Fig. 4a) and DLS (ESM Figure S2A), (ii) they are separated from lipoproteins and protein aggregates [42], and moreover, (iii) the overall procedure is performed within less than 30 min. The plasma exosomes were then analyzed by SPR. Our immunosensor chips proved again to be highly efficient to suppress nonspecific binding (ESM Figure S2B) and enabled the complete molecular biomarker screening (Fig. 4b). Although no conclusion regarding the clinical relevance of these data could be drawn, the particular expression pattern of this sample not only describes the membrane protein profile of a single population but also reflects the complexity of the entire blood exosomal population (Fig. 4c). Exosomes are mainly derived from blood cells (erythrocytes, leukocytes, and thrombocytes) and, to a lower extent, from other cell tissues (epithelial, nervous, muscle, or connective tissues). During the progression of cancer, exosomes derived from the diseased cells are released into biofluids, in particular blood, and thus modify the molecular profile of the exosomal population as they carry the molecular identity of the tumor [43]. Hence, the systematic implementation of our method on exosomes isolated from body fluids of both cancer patients and healthy donors, whose medical-related characteristics are known (e.g., age, sex, type, stage, and prognosis of the disease), would potentially link unique molecular signatures to particular types and stages of cancer. This novel strategy coupled to the isolation and purification of exosomes from body fluids would pave the way for a remote non-invasive and label-free diagnostic and prognostic test in cancer disease.

## Conclusion

We offer here an attractive alternative to the conventional label-based immunoassays for the rapid, multiplexed protein profiling of exosomes. While SPR techniques are generally used to detect small molecules or proteins, the large molecular mass of exosomes generate large signals even in SPR devices of simple optical design. Therefore, this technique offers enhanced detection sensitivity over competing standard techniques, circumventing the need of large sample volume. The combination of label-free and real-time detection of SPR provides fast analysis and minimizes tedious and time-consuming handling steps. We showed for the first time that exosomes from human blood could be purified and analyzed in less than 1 h using a commercially available instrument, offering a standard procedure for the development of a point-of-care patient sample analysis. The sensor architecture opens the door for clinicians to detect cancer-specific exosomes in a fully automated format using microliter volumes of blood. The immunosensor chip would provide easy, fast, minimally invasive, and continuous access to the state of tumors and therefore open a novel way in cancer diagnosis and personalized medicine without injuring the patients. The easy-to-prepare and easy-to-use sensor design offers the possibility to diagnose multiple cancer types with a single instrument.

## Electronic supplementary material

ESM 1 (PDF 1019 kb)
