# Supplementary material for: Molecular screening of cancer-derived exosomes by surface plasmon resonance spectroscopy
Source: Anal Bioanal Chem. 2015 Apr 30;407(18):5425–32. doi: 10.1007/s00216-015-8711-5 (PMC4477949; doi:10.1007/s00216-015-8711-5)
Supplement: Supplementary file 1 — (PDF 1019 kb) [file 216_2015_8711_MOESM1_ESM.pdf]

Analytical and Bioanalytical Chemistry

Electronic Supplementary Material

Molecular screening of cancer-derived exosomes by surface plasmon resonance spectroscopy

Luigino Grasso, Romain Wyss, Lorenz Weidenauer, Ashwin Thampi, Davide Demurtas, Michel Prudent, Niels Lion, Horst Vogel

Target protein	Name, Description	Antibody
CD9	CD9 antigen, a member of the tetraspanin family recognized as an exosomal protein marker.	eBioscience, clone eBioSN4
CD24	Signal transducer CD24, a heat stable antigen expressed in many tumour types.	eBioscience, clone eBioSN3
CD44	Hyaluronic acid receptor, a surface marker whose expression is related to the progression of many tumor types.	eBioscience, clone IM7
CD63	CD63 antigen, a member of the tetraspanin family recognized as an exosomal protein marker.	BioLegend, clone H5C6
CD326 (EpCAM)	Epithelial cell adhesion molecule, a membrane glycoprotein expressed in most normal human epithelial cells and overexpressed in most carcinomas. In breast cancer, its expression has been associated to poor prognosis.	BioLegend, clone 9C4
CD340 (HER2)	Human epidermal growth factor receptor 2, overexpression of this protein plays a major role in the development and progression of many cancers. An important biomarker for breast cancer therapy.	Sino Biological, clone 8B5D4C1
IgG1 (rat)	Immunoglobulin G1, used here as a negative control.	BioLegend, clone MRG1-58

Table S1: List of target proteins and their respective biotinylated antibodies used in the SPR experiments.

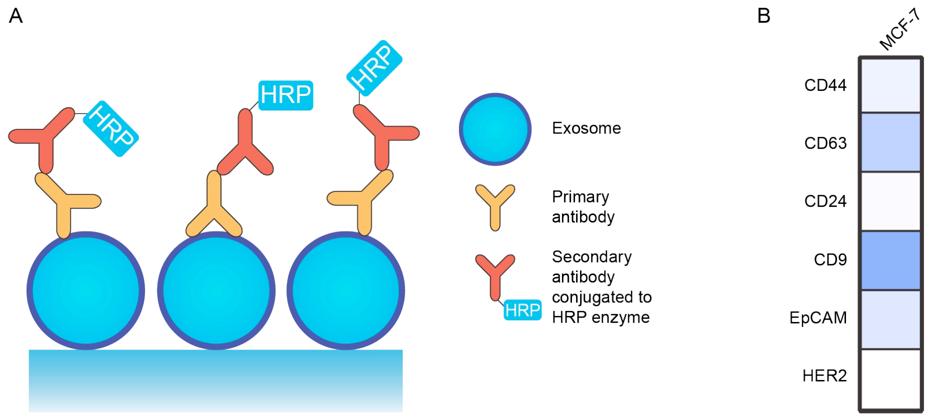

Figure S1. Molecular screening of exosomes by ELISA. (A) Scheme of molecular screening by ELISA. After exosome adsorption on the surface of a 96-well plate, primary antibodies specifically bind exosomal surface antigens. The binding of secondary antibodies conjugated to HRP enzymes is then revealed with TMB, causing the development of a blue color. (B) Molecular profiling of breast cancer and exosomal biomarkers in MCF-7 exosomes. Data were corrected with negative controls (without exosomes).

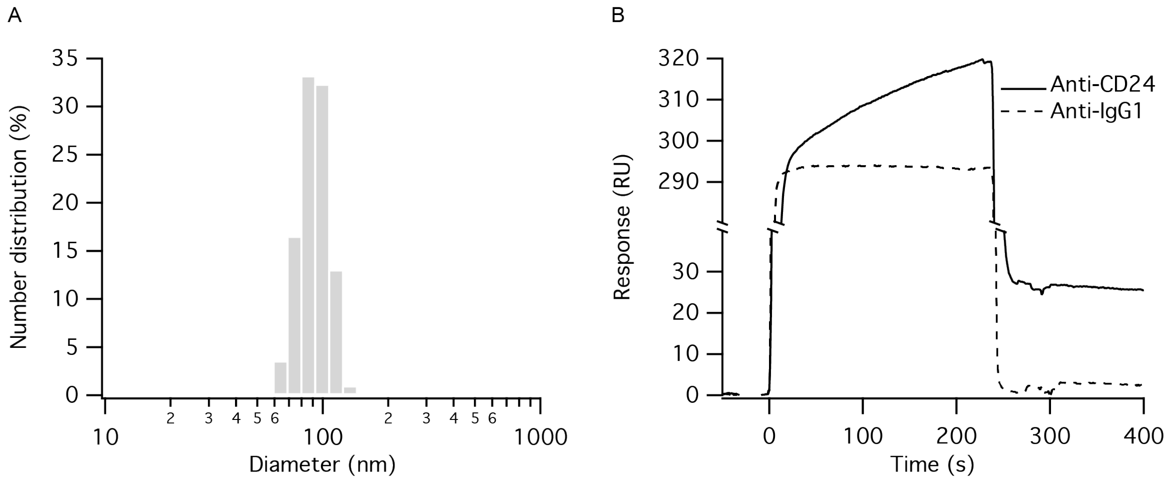

Figure S2. Exosomes from plasma. (A) Size distribution of plasma exosomes obtained by DLS. (B) Specific detection of plasma exosomes with biotinylated anti-CD24 antibodies. Anti-rat IgG1 was used as a negative control.
